# Improving acute kidney injury alerts in tertiary care by linking primary care data: An observational cohort using routine care data

**DOI:** 10.1177/20552076241271767

**Published:** 2024-08-18

**Authors:** Huibert-Jan Joosse, Wouter Tiel Groenestege, Robin WM Vernooij, Mark CH De Groot, Imo E Hoefer, Wouter W van Solinge, Maarten B Kok, Saskia Haitjema

**Affiliations:** 1 Central Diagnostic Laboratory, University Medical Center Utrecht, Utrecht University, Utrecht, The Netherlands; 2 Department of Nephrology and Hypertension, University Medical Center Utrecht, Utrecht University, Utrecht, The Netherlands; 3 Julius Center for Health Sciences and Primary Care, University Medical Center Utrecht, Utrecht University, Utrecht, The Netherlands; 4 Saltro BV, Unilabs Netherlands, Utrecht, The Netherlands

**Keywords:** Acute kidney injury, data linkage, diagnostic accuracy, health informatics, kidney

## Abstract

**Objective:**

Acute kidney injury (AKI) is easily missed and underdiagnosed in routine clinical care. Timely AKI management is important to decrease morbidity and mortality risks. We recently implemented an AKI e-alert at the University Medical Center Utrecht, comparing plasma creatinine concentrations with historical creatinine baselines, thereby identifying patients with AKI. This alert is limited to data from tertiary care, and primary care data can increase diagnostic accuracy for AKI. We assessed the added value of linking primary care data to tertiary care data, in terms of timely diagnosis or excluding AKI.

**Methods:**

With plasma creatinine tests for 84,984 emergency department (ED) visits, we applied the Kidney Disease Improving Global Outcome guidelines in both tertiary care-only data and linked data and compared AKI cases.

**Results:**

Using linked data, the presence of AKI could be evaluated in an additional 7886 ED visits. Sex- and age-stratified analyses identified the largest added value for women (an increase of 4095 possible diagnoses) and patients ≥60 years (an increase of 5190 possible diagnoses). We observed 398 additional visits where AKI was diagnosed, as well as 185 cases where AKI could be excluded. We observed no overall decrease in time between baseline and AKI diagnosis (28.4 days vs. 28.0 days). For cases where AKI was diagnosed in both data sets, we observed a decrease of 2.8 days after linkage, indicating a timelier diagnosis of AKI.

**Conclusions:**

Combining primary and tertiary care data improves AKI diagnostic accuracy in routine clinical care and enables timelier AKI diagnosis.

## Introduction

Acute kidney injury (AKI) is an abrupt decrease in glomerular filtration, which is present in 10% to 20% of hospitalized patients,^1,2^ whereas the incidence is even higher (50%) in patients in the intensive care unit.^1^ Incidence of AKI is expected to increase in the future, due to a higher prevalence of its risk factors, such as cardiovascular disease and diabetes.^3^ Given the estimated 10% increase of mortality risk in hospitalized patients and extended length of hospital stay,^2^ it is important to diagnose AKI as soon as possible.^4^ Nevertheless, AKI is easily missed, since it often co-exists with other morbidities and can have pre-, post-, or intrinsic renal causes. AKI is not only associated with a worse short-term prognosis, since patients with AKI have a substantially increased risk of progressing to chronic kidney disease (CKD) and end-stage kidney disease, which subsequently are associated with a high mortality and morbidity rate.^5^ Finally, earlier studies showed that diagnosis is missed in (subgroups of) elderly patients^6,7^ and women.^8^

In order to better identify patients with AKI, we recently implemented an AKI e-alert in the University Medical Center Utrecht (UMCU), based on the Kidney Disease Improving Global Outcome (KDIGO) guidelines for AKI. This implementation resulted in an increase in follow-up plasma creatinine measurements and higher rates of cessation of nephrotoxic medicine after an AKI alert,^9^ signifying increased awareness. However, this AKI e-alert is currently limited to tertiary care data. Since the diagnosis of AKI is dependent on the change of plasma creatinine during a specific time frame, including historical data from primary and secondary care may help to diagnose or exclude AKI more accurately. Linking data from other sources to tertiary care data might add clinical value to the routine care process, as it will increase the available information for each patient during this timeframe and therefore possibly improve the diagnostic accuracy of AKI identification.

An important aspect of adding value to healthcare by means of data linkage is a large degree of overlap between the sources. In the Utrecht region of the Netherlands, laboratory testing for primary care is primarily performed by Saltro (Unilabs Eerstelijnsdiagnostiek B.V.). Considering the potential of these data to benefit clinical diagnosis, we investigated to what degree linked data of plasma creatinine from Saltro and tertiary care data from the UMCU improve the accuracy of the AKI e-alert at the UMCU emergency department (ED) when compared to using tertiary care-only data. To better understand the added value of using linked data, we performed subgroup analyses for both sex and age.

## Methods

### Data

We retrieved data for all patients who presented at the ED of the UMCU between June 2011 and December 2018. For each visit, we retrieved plasma creatinine results from the UMCU and linked them to plasma creatinine results available at Saltro (Unilabs Eerstelijnsdiagnostiek B.V.) for the same patients. Data from both sources were pseudonymized before exchange, and another layer of pseudonymization was added after linkage in order to comply with general data protection regulations. We selected every measurement from 370 days before to up to 30 days after the presentation at the ED for each patient. The study was conducted in accordance with the Declaration of Helsinki.

### Applying KDIGO guidelines

We applied the KDIGO guidelines per patient for all available plasma creatinine results. These guidelines are based on the increase of creatinine in blood in a specific timeframe.^10^ In brief, if there is any increase in excess of 27 µmol/L of plasma creatinine in the last 48 h or an increase of plasma creatinine with a ratio of ≥1.5 with any previous plasma creatinine measurement in the last 365 days, a patient was labeled as having AKI. For visits where plasma creatinine was measured at the UMCU at ED visit, we applied the guidelines twice: once on tertiary care-only data and once on the linked data (Saltro and UMCU).

Based on the date and time of presentation at the ED, we defined the creatinine result at ED presentation as the plasma creatinine measurement that was closest to the ED visit in time, with a maximum difference of 24 h either before or after the reported ED visit date time and used the corresponding AKI diagnosis in our analyses. If there was a creatinine result at the ED presentation but no baseline could be established in either Saltro or UMCU data, we labeled the visit as “no baseline.” If there was no plasma creatinine measurement within 24 h of ED visit, we excluded the visit from our analyses.

### Analysis

We compared the number of ED visits where AKI was identified using the linked data and tertiary care-only data. To assess whether linking our data sources improved AKI diagnosis or exclusion, we assessed the number of extra AKI cases using primary care data (reduction of false negatives), as well as the number of cases that were labeled as AKI when only using tertiary care data, but not when using primary care data (reduction of false positives), respectively. We also assessed the time to the baseline plasma creatinine value on which the AKI criteria were met (timelier diagnosis of AKI). We performed subgroup analyses for sex and age at ED visits (age <60 and age ≥60; based on median age (59)) to assess the added value of linking data in these subgroups.

We compared the number of diagnoses using the chi-square test and analyzed the difference in time between baseline and ED visits with the use of the Wilcoxon rank sum test, considering the non-normal distribution of these data.

### Software

Analyses were performed using the Python programming language (version 3.11), in conjunction with the *pandas* (version 1.5.3), *numpy* (version 1.24.2), and *tableone* (version 0.7.2) packages. Visualizations were made with the *seaborn* (version 0.12.2) and *matplotlib* (3.7.0) packages.

## Results

**Figure 1.**
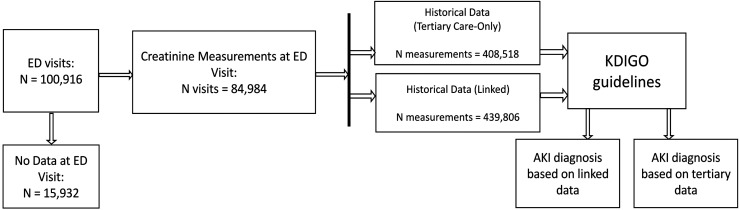
Flowchart displaying the followed workflow during data selection and analysis.

We retrieved data for 100,916 consecutive UMCU ED visits from 51,302 patients between June 2011 and December 2018. For 15,932 visits, there was no plasma creatinine measurement available, resulting in 84,984 visits from 47,271 patients with a creatinine measurement that was used for analysis (Figure 1). The ED visits were equally distributed among the sexes (46.1% and 53.9% visits by female and male patients, respectively) and patients had a median age of 59.0 [interquartile range: 44.0–71.0] at the time of their visit. For 12,987 (27.5%) patients, we obtained data from both primary and tertiary care within 370 days prior and 30 days after ED presentation (Table 1).

**Table 1.** Characteristics of the ED visits with plasma creatinine measurements at UMCU between June 2011 and December 2018 for the overall population, visits with only tertiary care data available, and visits with data from both sources.

		Overall	Visits with data from tertiary care only	Visits with data from both sources
Number of visits, *n*		84,984	64,078	20,906
Unique patients, *n*		47,271	34,284	12,987
Sex, *n* (%)	Female	39,251 (46.1)	24,621 (44.7)	14,630 (48.7)
	Male	45,855 (53.9)	30,445 (55.3)	15,410 (51.3)
Age, median [Q1, Q3]		59.0 [44.0, 71.0]	55.0 [39.0, 67.0]	66.0 [53.0, 76.0]
Creatinine (µmol/L), median [Q1, Q3]		75.0 [62.0, 96.0]	75.0 [62.0, 93.0]	78.0 [63.0, 104.0]

ED: emergency department; UMCU: University Medical Center Utrecht; *n*: number; Q: quartile.

### Comparison between using linked versus tertiary care-only data

In total, 2639 visits (3.11%) were diagnosed with AKI, based on tertiary care-only data (Table 2). Linkage increased the number of diagnosed AKI cases (*n* = 211, 8.00%) as well as the cases where a baseline was available for excluding AKI (*n* = 7655, 16.50%; Table 2). As a result, we observed a decrease in visits where no baseline could be established (*n* = 7866, −21.87%). We observed no significant overall decrease in time between visit and baseline for cases with AKI. However, we did observe a significant decrease from 26.1 to 23.3 days (delta = 2.8 days, *p* < 0.001) for cases with AKI as diagnosed in both tertiary care-only and linked data, indicating a timelier diagnosis of AKI.

**Table 2.** AKI characteristics of the ED visits with and without using primary care data (number of visits = 84,948).

		Tertiary care-only data (% of Visits)	Linked data (% of visits)	Difference (% of tertiary care-only data)	*p*
KDIGO guidelines diagnosis	AKI	2639 (3.11)	2850 (3.36)	+211 (8.00)	<0.001
	No AKI	46,380 (54.57)	54,035 (63.60)	+7655 (16.50)	
	No baseline	35,965 (42.32)	28,099 (33.04)	−7866 (−21.87)	
Time to baseline (days), median [Q1, Q3]		28.4 [8.9, 83.0]	28.1 [7.8, 82.7]	−0.3	0.260

AKI: acute kidney injury; ED: emergency department; KDIGO: Kidney Disease Improving Global Outcome; Q: quartile.

Table 3 compares the AKI cases that were identified using tertiary care-only data versus using linked data and the overlap of AKI cases between the two. We found 185 (7.01% of AKI cases based on tertiary care-only data) visits where AKI was identified in the tertiary care data only, and 398 (15.08% of AKI cases based on linked data) visits were identified as AKI using linked data only (Table 3).

**Table 3.** Comparison of acute kidney injury (AKI) identifications in the linked data versus tertiary care data only.

		Linked data		
		AKI	No AKI	No baseline
Tertiary care-only data
	AKI	2454	185	0
	No AKI	58	46,322	0
No baseline	340	7546	28,079	

### Sex-specific results

**Figure 2.**
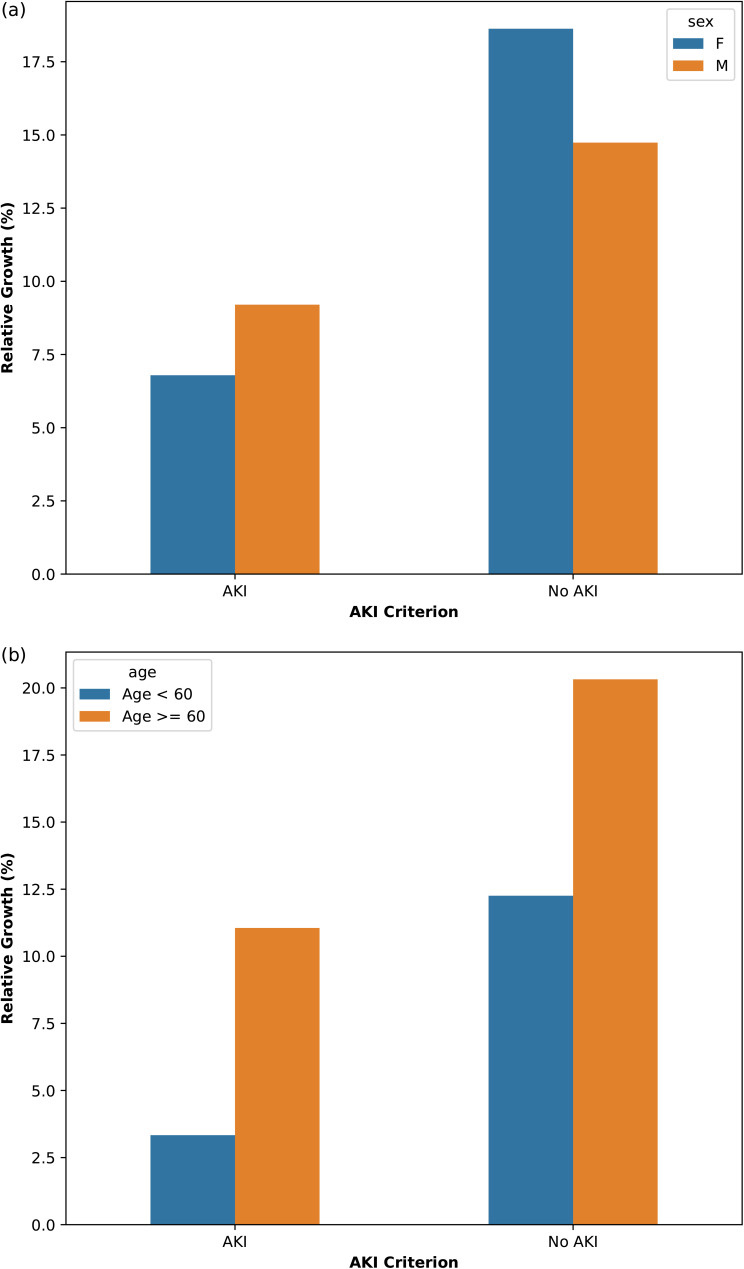
The increase of diagnosed emergency department (ED) visits after linking primary and tertiary data, stratified according to sex (a) and age (b).

The sex-stratified subgroup analyses showed a larger relative change for the diagnosis of AKI for women than for men (24.93% increase in total visits where a diagnosis was possible for women vs. 19.39% for men) (Table 4). There was a larger increase of diagnosed AKI cases for men compared to women (129 vs. 84; 9.20% vs. 6.59%, respectively), and there was a higher relative increase of visits where AKI was excluded for women compared to men (4011 vs. 3662; 18.62% vs. 14.74%, respectively) (Figure 2(a)). Moreover, we observed an overall decrease of 2 days between baseline measurement and ED measurement for women (*p* = 0.091), compared with only −0.2 days for men (Table 4). Considering cases for which AKI was diagnosed with and without primary care data, we observed a significant decrease for both women and men. The time difference between baseline and visit for these cases was reduced from 27.2 to 24.9 days for women (*p* < 0.001) and from 24.4 to 22.7 days for men (*p* < 0.001).

**Table 4.** AKI characteristics of the ED visits with and without using primary care data, stratified according to sex.

			Tertiary care-only data (% of visits)	Linked data (% of visits)	Difference (% of tertiary care-only data)	*p*
Women	KDIGO guidelines diagnosis	AKI	1237 (3.16)	1321 (3.37)	+84 (6.59)	<0.001
		No AKI	21,532 (54.94)	25,543 (65.18)	+4011 (18.62)	
		No Baseline	16,421 (41.90)	12,326 (31.45)	−4095 (−24.93)	
	Time to baseline (days), median [Q1, Q3]		30.0 [9.2, 88.4]	28.0 [7.2, 82.9]	−2.0	0.091
Men	KDIGO guidelines diagnosis	AKI	1402 (3.06)	1531 (3.34)	+129 (9.20)	<0.001
		No AKI	24,848 (54.26)	28,510 (62.26)	+3662 (14.74)	
		No baseline	19,544 (42.68)	15,753 (34.40)	−3791 (−19.39)	
	Time to baseline (days), median [Q1, Q3]		27.6 [8.3, 77.9]	27.8 [8.1, 81.7]	−0.2	0.967

AKI: acute kidney injury; ED: emergency department; KDIGO: Kidney Disease Improving Global Outcome; Q: quartile.

### Age-related results

The age-stratified subgroup analyses showed a larger increase in diagnosable visits for older patients (33.20%) than for younger patients (13.25%) (Table 5). There was a larger increase in the number of AKIs and non-AKIs for the older patient population (Figure 2(b)). We observed no significant overall decrease in time between baseline and ED visits for both patient groups (−0.5 days for patients <60; −0.9 days for patients age ≥60) (Table 5). As with the overall analysis, we did observe a decrease in time between baseline and ED visits for cases where AKI was diagnosed in both the tertiary care-only data and linked data sets. The time between baseline and visit decreased for both age groups: from 27.2 to 24.2 days for patients aged 60 and older (*p* < 0.001), whereas for patients under 60, this time decreased from 23.6 to 22.1 days (*p* < 0.001).

**Table 5.** AKI characteristics of the ED visits with and without using primary care data, stratified according to age.

			Tertiary care-only data (% of visits)	Linked data (% of visits)	Difference (% of tertiary care-only data)	*p*
Age <60	KDIGO guidelines diagnosis	AKI	1020 (2.37)	1054 (2.45)	+34 (+3.33)	<0.001
		No AKI	21,719 (50.42)	24,381 (56.60)	+2662 (+12.25)	
		No Baseline	20,334 (47.21)	17,638 (40.95)	−2696 (−13.25)	
	Time to baseline (days), median [Q1, Q3]		25.4 [7.2, 76.1]	24.9 [7.1, 77.5]	−0.5	0.873
Age ≥60	KDIGO guidelines diagnosis	AKI	1619 (3.86)	1798 (4.29)	+179 (+11.05)	<0.001
		No AKI	24,661 (58.84)	29,672 (70.80)	+5011 (+20.32)	
		No baseline	15,631 (37.30)	10,441 (24.91)	−5190 (−33.20)	
	Time to baseline (days), median [Q1, Q3]		29.9 [9.7, 87.3]	29.0 [8.2, 84.9]	−0.9	0.175

AKI: acute kidney injury; ED: emergency department; KDIGO: Kidney Disease Improving Global Outcome; Q: quartile.

## Discussion

To assess the added value of linking data for the diagnosis and exclusion of AKI, we compared the diagnosis and exclusion of AKI at the UMCU ED with tertiary care-only data to linked data. In our study, including 84,984 ED visits, we found a 21**.**87% increase in visits where a baseline was present to facilitate diagnosis or exclusion of AKI. Moreover, we observed a reduction of false negatives (*n* = 58; 2**.**20% of AKI cases based on linked data), an increase in positive cases (*n* = 340; 12.88% of AKI cases based on linked data), and also a reduction of false positives due to a more recent baseline (*n* = 185; 7.01% of AKIs identified with tertiary care-only data) (Table 3). We found no overall decrease in time between baseline and visits for AKI cases after data linkage (Table 2). However, we do find significant decreases from 26.1 to 23.3 days for cases where AKI was identified in both the tertiary care-only data and linked data, indicating more timely AKI diagnosis.

Subgroup analyses show the highest increase of visits where a diagnosis or exclusion was possible for both women and patients ≥60 years. Considering women and elderly patients are underdiagnosed and AKIs are easily missed in these patients,^6–8^ adding more data to provide more insight into these subgroups might help improve AKI diagnosis. The higher added value for women and patients ≥60 years can be explained by higher primary care usage from women and elderly patients.^11–13^ For sex, we observed a larger relative increase in AKI cases for men after data linkage. In general, AKI is more often diagnosed in men compared to women, for example, because of higher muscle mass and thus higher plasma creatinine levels in men. Another explanation is the nephroprotective role of estrogens.^8,14,15^ However, our observations might also be a result of underdiagnosis in women, as a result of lower baseline creatinine. As a result, the absolute increase of 27 µmol/L within 48 h is a substantially higher percentage increase for women and patients ≥60.^15^ This can also partly explain the larger time interval between AKI and baseline measurements for both women and patients ≥60 years**.** In this study, AKI diagnosis was solely based on plasma creatinine measurement, irrespective of sex and other possible contributing factors.

For visits where AKI was identified in both linked and tertiary care-only data, we observed a significant decrease in time to the baseline. This underlines the importance of combining data from different sources, as combining data can, for example, identify the timeframe in which AKI developed. Timely identification in a routine care setting might improve the outcome of the patient since timely management can prevent further deterioration of kidney function. For cases where an AKI was excluded, monitoring and management of AKI might no longer be necessary. This can, for example, mean that valuable yet nephrotoxic medications are not discontinued.

Utilizing different data sources across the healthcare domain already showed promise in modeling efforts in skin pathology, macular degeneration, and breast cancer detection, resulting in improved accuracy and generalizability of predictive models.^16^ Another way of data sharing through decentralized storage has shown promise in genomics and medical imaging in combination with federated learning.^17^ Importantly, data sharing in healthcare is primarily important to achieve efficient and accurate diagnosis in healthcare and can help prevent redundant testing.^18^ Therefore, data sharing and linkage in routine healthcare might require a more centralized system or platform, since data should be available to all relevant healthcare providers throughout the healthcare system. Moreover, sharing data must be performed with the privacy and integrity of patients in mind, as data sharing can become problematic for patients when third parties have access to their data, e.g. private insurance companies.^16,19^ Setting up robust data-sharing initiatives should result in secure and usable data, as well as mitigation of any risk that could be harmful to patients.^20^ In recent years, the European Commission has proposed a European Health Data Space (EHDS) to provide a framework in which data can safely be exchanged, and also where patients can have more agency on their data.^21^ Developments such as the EHDS can help set up more efficient data sharing and transfer in both research and clinical care, while complying with the GDRP. Initiatives can further incentivize utilization of historical patient data from different healthcare settings to improve diagnostic accuracy, as we show in our study.

However, establishing a data-sharing framework can come at an expense. For example, healthcare data can be incompatible between healthcare providers.^22,23^ Incompatibility of data requires precious time of data experts to ensure harmonization and interoperability of patient data. However, our study shows that when data are shared, the diagnostic accuracy for AKI can substantially improve, possibly leading to fewer wrongful admissions and discharges. However, the true extent of the cost-effectiveness remains to be measured. However, evidence shows the massive costs of treating AKI in the United Kingdom,^24^ and linking creatinine data across the healthcare domain can be an important step to better respond to AKI development in patients.^25^ The implementation of an AKI alert based on primary and secondary care data in the United Kingdom already resulted in increased hospitalization rates within 7 days of AKI and improved creatinine monitoring.^26^ Thus, improving creatinine monitoring can help increase clinical response to AKI and possibly prevent the development of AKI in at-risk populations. Prevention of AKI is an important step in diminishing the large economic burden of in-hospital AKI.^27^

There are some limitations to our study. Firstly, we have no access to historical patient data from secondary care. Adding this source of data would likely result in even more visits where we can rightfully apply the KDIGO guidelines to identify AKI. Secondly, we used the last known creatinine value as a baseline, rather than the lowest, the mean, or the median, for the time window between 7 and 365 prior to ED presentation. We previously published the assessment of a “baseline” creatinine value in routine clinical care.^28^ In this study, AKI prevalence remained similar when using the last known creatinine value, or the median or mean over this time period. However, a substantial increase in prevalence was observed using the lowest known value, which in turn can lead to overestimation of AKI prevalence.^28^ We therefore deem the prevalence of AKI in our study to be representable for the patient population at the UMCU. Finally, we have not investigated the presence of CKD or acute CKD, as the scope of this research is based on AKI. Future research could entail the identification of CKD. However, based on the descriptive data of our study (Table 1), we believe that the presence of CKD is limited within our patient selection.

## Conclusion

In our study, we investigated the impact of linking primary and tertiary care data for diagnosis and exclusion of AKI, based on the KDIGO guidelines in ED visits. We found that we are able to diagnose or exclude more visits for AKI in general and diagnose more visits correctly as having AKI (decreased false negatives). Interestingly, we also observed visits where we could exclude AKI (decreased false positives). Finally, we found a shorter time between visit and baseline for patients where AKI was identified in linked data compared to tertiary care-only data, indicating a timelier AKI diagnosis. Considering these findings, more effort should be taken to combine data from different care settings, as this may help increase diagnostic accuracy and subsequent treatment for ED patients with and without AKI.
